# Azygos Vein Patch for Enlarging Anastomosis of Ascending Aorta and Neoaorta After Norwood Procedure

**DOI:** 10.1016/j.atssr.2022.11.016

**Published:** 2022-12-01

**Authors:** Shiori Kimura, Yasutaka Hirata, Miyuki Shibata, Minoru Ono

**Affiliations:** 1 Faculty of Medicine, The University of Tokyo, Tokyo, Japan; 2 Department of Cardiac Surgery, The University of Tokyo Hospital, Tokyo, Japan

## Abstract

Pericardium and polytetrafluoroethylene are popular patch materials, although they have some deficits in use for thin and small vessels. We present a case of post-Norwood hypoplastic left heart syndrome that required enlargement of the anastomosis between the native ascending aorta and the neoaorta at the time of a bidirectional Glenn procedure. The azygos vein was a useful material for patch augmentation of a hypoplastic ascending aorta.

Hypoplastic left heart syndrome (HLHS) is a structural defect and insufficiency in the capacity of the left ventricle to provide systemic perfusion. Today, 3 stages of surgical reconstruction provide a pathway for survival, and approximately 70% to 80% of patients who undergo operation for HLHS survive up to 20 years thereafter.^1^

In Japan, autologous or bovine pericardial patches and polytetrafluoroethylene patches are commonly used in congenital heart surgery instead of homograft; however, they carry a relatively high risk of restenosis or calcification. ^2^^,^^3^ We here report the use of the azygos vein as a patch for plasty of a hypoplastic ascending aorta, which ultimately restored cardiac function.

A term infant with a birth weight of 2.8 kg and a prenatal diagnosis of HLHS was referred to us. Postnatal echocardiography demonstrated HLHS with aortic and mitral atresia and a large atrial septal defect with adequate mixing of blood at the atrial level. The ascending aorta measured 1.5 mm in diameter. The patient underwent neonatal bilateral pulmonary artery banding followed by the Norwood procedure at 7 weeks of age. After uneventful recovery, she was discharged on postoperative day 41 with a saturation around 80% and a brain natriuretic peptide (BNP) level of 77 pg/mL (Figure 1).

**Figure 1:**
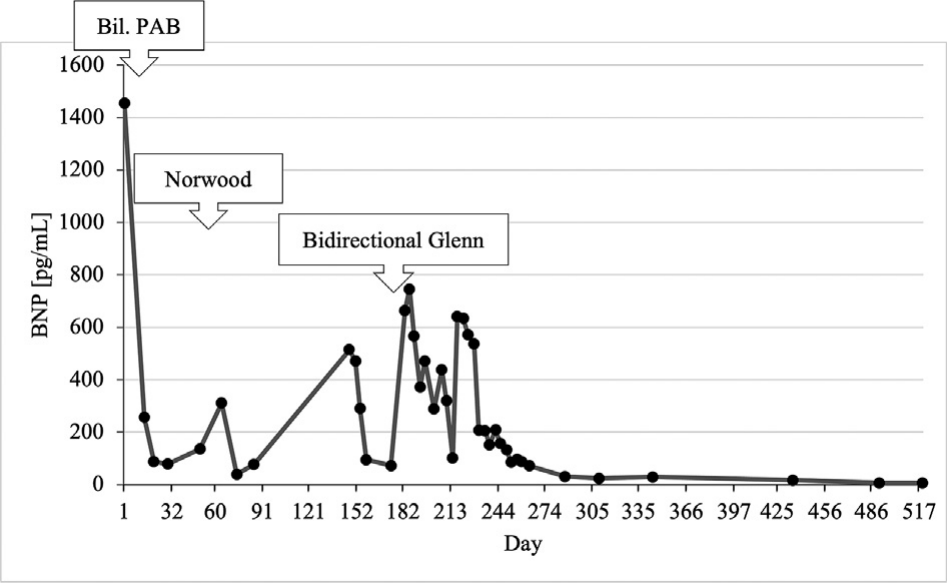
Time course of plasma brain natriuretic peptide (BNP) levels. Preoperative brain natriuretic peptide level of 514 pg/mL decreased to 17 pg/mL at 6-month follow-up after bidirectional Glenn procedure. (Bil. PAB, bilateral pulmonary artery banding.)

During follow-up, however, her saturation level decreased to 65% to 69%, and she was admitted for close examination. On admission, her BNP level was high at 514 pg/mL (Figure 1), and echocardiography showed poor contraction of the right ventricle. Contrast-enhanced cardiac computed tomography (CT) scan revealed stenosis at the anastomosis between the native ascending aorta and the neoaorta (Figure 2A). Holter electrocardiography showed unspecific ST-T changes and recurrent episodes of strain T waves. Therefore, a decision was made to augment the hypoplastic ascending aorta during a bidirectional Glenn procedure, with the aim of alleviating the probable cardiac ischemia.

**Figure 2:**
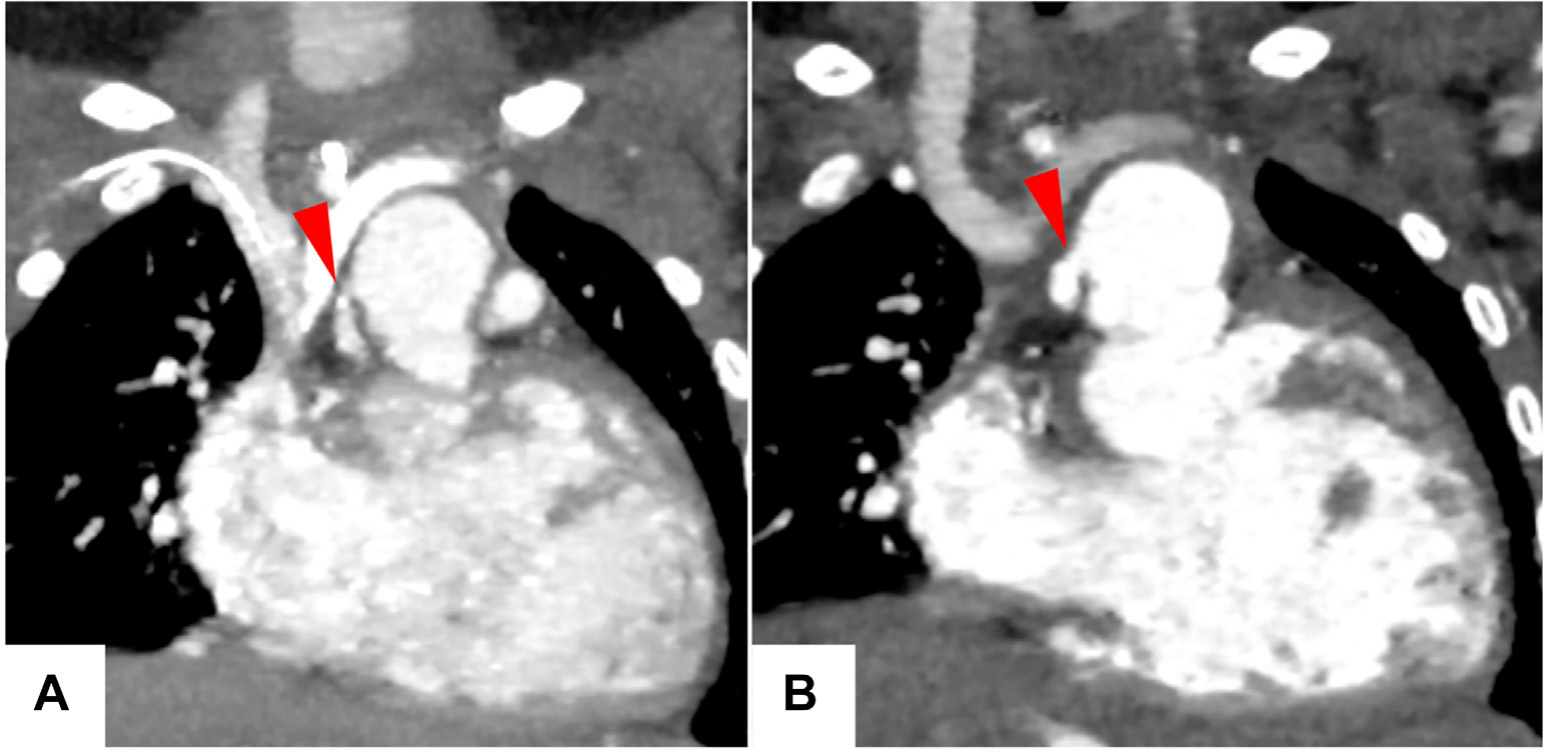
(A) Preoperative computed tomography image showing stenosis (arrowhead) at the anastomosis between native ascending aorta and neoaorta. (B) Postoperative computed tomography image showing its resolution (arrowhead).

The operation was performed at 6 months of age. After midline sternotomy, cardiopulmonary bypass (CPB) was established with arterial cannulation at the neoaorta and venous cannulation at the right atrium. The right ventricle–pulmonary artery shunt was taken down by transecting the conduit and closing both ends. The azygos vein was ligated behind the superior vena cava, and 8 mm of the vein wall was harvested to create a patch.

As aortoplasty was difficult with aortic cross-clamping, this part of the procedure was performed with hypothermic circulatory arrest; after establishment of venous cannulation at the superior vena cava, circulatory arrest was achieved at a rectal temperature of 20 °C, and myocardial protection was provided with antegrade St. Thomas crystalloid cardioplegia. The neoaorta was incised along the suture, and the site of anastomosis of the native ascending aorta to the native pulmonary artery could be observed. It was stenotic in a slitlike fashion (Figure 3A); hence, the incision line was extended outward to open the stenosis (Figure 3B). Here, a patch made of azygos vein incised longitudinally was sutured (Figure 3C), and the remaining part was closed. After air removal and resumption of circulation, the superior vena cava was cut before its insertion into the right atrium and sutured to the right pulmonary artery to complete the bidirectional Glenn procedure. The patient was weaned off CPB; postoperative central venous pressure was 15 to 18 mm Hg, and saturation was 85% with nitric oxide 20 ppm and fraction of inspired oxygen 1.0. CPB time was 168 minutes, and circulatory arrest time was 41 minutes. The patient was transferred to the pediatric intensive care unit and extubated on postoperative day 3. After gradual recovery, the patient was discharged on postoperative day 91 with a BNP level of 72 pg/mL. Predischarge CT scan was good; stenosis of the native aorta had resolved (Figure 2B). The BNP level at 6-month follow-up was 17 pg/mL, within the normal range (Figure 1).

**Figure 3:**
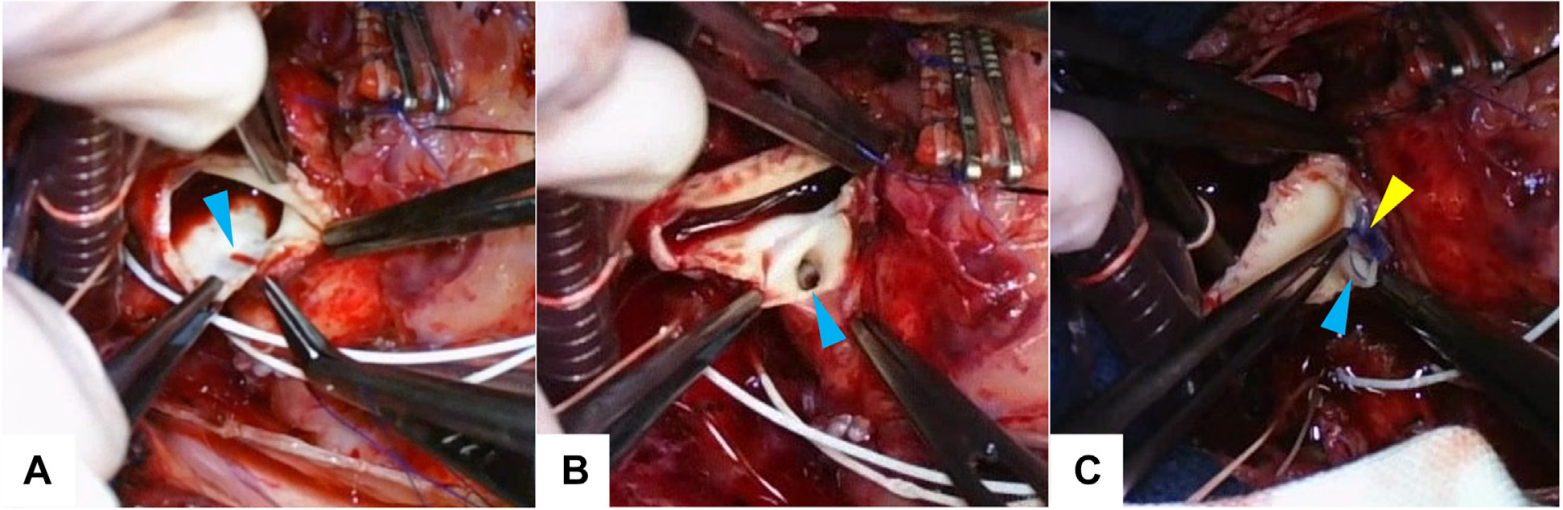
(A) Slitlike stenosis (arrowhead) of native ascending aorta. (B) Stenosis released (arrowhead) by extending the incision line. (C) Suturing the azygos vein patch (yellow arrowhead) to the native ascending aorta (blue arrowhead).

## Comment

We found 2 important clinical issues: azygos vein patch can be useful in thin ascending aorta plasty; and reduced cardiac function after the Norwood procedure may be restored by ascending aorta plasty and relief of myocardial ischemia.

First, azygos vein patch can be useful for enlargement of a hypoplastic ascending aorta. Classically, patches cut out from pulmonary bifurcation homografts have been used for aortic arch reconstruction, but they are prone to calcification, and their availability is limited in Japan. Autologous pericardium has been shown to have a significantly lower recoarctation rate. Bovine or equine pericardium can also be used, which has been reported to have a higher incidence of recoarctation compared with homografts and autologous pericardium.^2^ Use of a polytetrafluoroethylene patch for aortic arch reconstruction has been described in previous studies, in which patients with ascending aorta of at least 3 mm in diameter were selected because the authors deemed that this material is too stiff and thick to be used in thin and small native aortas.^3^ In our case, however, the native ascending aorta was 1.5 mm. Accordingly, the wall of the patch material should be sufficiently thin to achieve a good anastomosis. An azygos vein patch or graft has been reported to be useful in surgical coronary angioplasty for congenital left coronary artery obstruction.^4^ Thus, the azygos vein patch is probably thin enough, and in addition, the azygos vein is the vessel routinely ligated in the bidirectional Glenn procedure, making this material the most suitable for expansion of the ascending aortic anastomosis at this stage.

Second, cardiac function, which is reduced after the Norwood procedure, can be restored by enlarging the ascending aorta to alleviate myocardial ischemia. During the interstage period (between the Norwood and the bidirectional Glenn procedures), even the most stable patients are at risk for acute hemodynamic decompensation. Death rate during this period ranges from 2% to 16% according to previous studies. Various anatomic lesions, including restrictive atrial septal defect, stenosis or obstruction of the conduit or aortic arch and pulmonary arteries, and tricuspid regurgitation, have been associated with interstage death.^5^ In our case, signs of heart failure were detected after discharge following the Norwood operation. Cardiac CT scan revealed stenosis of the native ascending aorta, and Holter electrocardiography showed ST-T changes, suggesting that decreased coronary artery perfusion was responsible for the cardiac function decline. Although the bidirectional Glenn procedure itself reduces volume load on the ventricle, contributing to less workload, early detection and surgical intervention of the stenosis seem to have resulted in excellent clinical outcome without signs of heart failure or elevated BNP levels at follow-up.

In conclusion, an azygos vein patch can be useful for plasty of a hypoplastic ascending aorta, and reduced cardiac function after the Norwood procedure can be restored by augmenting the ascending aorta to relieve myocardial ischemia. The use of the azygos vein as a patch for plasty should be considered when it is performed at the same time as the bidirectional Glenn procedure.
